# Renewal of the Scope of Medical Sciences

**DOI:** 10.3390/medsci3010001

**Published:** 2015-03-06

**Authors:** Yu-Jia Chang

**Affiliations:** Graduate Institute of Clinical Medicine, Taipei Medical University, Taipei, Taiwan; E-Mail: r5424012@tmu.edu.tw; Tel.: +886-2-27361661 (ext. 3027)

From time to time, it is useful to reassess a journal’s purpose, and position it in a way that it can make a significant and unique contribution to the scientific literature. I have recently completed the task of adjusting the scope of *Medical Sciences* (ISSN 2076-3271; http://www.mdpi.com/journal/medsci), and I believe it is now in an even better position to publish significant work that will be of use and interest to scholars and clinicians within the medical science field.

*Medical Sciences* focuses on the contribution of basic science to medicine. The journal will publish original research and comprehensive reviews in the following areas, as related to this aim:

- biochemistry/molecular biology
- bioinformatics and systems biology
- genetics/genomics
- pathology and physiology
- biostatistics
- epidemiology
- immunology and microbiology
- neuroscience
- pharmacology and pharmacogenomics
- toxicology

We are always interested to hear feedback from authors and receive proposals for potential papers or Special Issues. I would be happy to make comments on such proposals to ensure a good chance of success. You can contact either myself or the editorial office.

With the valuable support of the Editorial Board, I am confident of the continued growth and success of *Medical Sciences*.

